# Supplementary material for: The pronounced lung lesions developing in LATY136F knock-in mice mimic human IgG4-related lung disease
Source: PLoS One. 2021 Mar 4;16(3):e0247173. doi: 10.1371/journal.pone.0247173 (PMC7932159; doi:10.1371/journal.pone.0247173)
Supplement: S1 File — (DOCX) [file pone.0247173.s001.docx]

S1 File.

Supporting information of Fig. 3B

|  | LAT CD138 (mean ± S.D.) | WT CD 138 (mean ± S.D.) |
| --- | --- | --- |
| 4W | 51.8 ± 4.19 | 0.00 ± 0.00 |
| 6W | 120.83 ± 24.38 | 5.33 ± 4.99 |
| 10W | 309.67 ± 55.15 | 16.67 ± 5.44 |
| 20W | 170.83 ± 32.81 | 12.33 ± 5.10 |

|  | LAT IgG1 (mean ± S.D.) | WT IgG1 (mean ± S.D.) |
| --- | --- | --- |
| 4W | 38.33 ± 16.44 | 0.00 ± 0.00 |
| 6W | 78.67 ± 7.54 | 0.67 ± 0.94 |
| 10W | 240.83 ± 81.23 | 0.67 ± 0.94 |
| 20W | 108.67 ± 45.06 | 2.00 ± 1.41 |

Supporting information of Fig. 4

Achcroft score

|  | LAT (mean ± S.D.) | WT (mean ± S.D.) |
| --- | --- | --- |
| 4W | 0.38 ± 0.41 | 0.00 ± 0.00 |
| 6W | 1.06 ± 0.63 | 0.00 ± 0.00 |
| 10W | 2.22 ± 1.03 | 0.70 ± 0.68 |
| 20W | 5.43 ± 0.90 | 0.70 ± 0.75 |

Supporting information of Fig. 5

BALF findings

|  |  |  | **LAT (n=8)**  (mean ± S.D.) | **WT (n=7)**  (mean ± S.D.) |  |
| --- | --- | --- | --- | --- | --- |
| Cell differenciation | TCC | (×10^5^/μL) | 11.9 ± 8.09 | 2.00 ± 0.40 | p = 0.005 |
|  | MΦ | (×10^5^/μL) | 1.82 ± 1.76 | 1.76 ± 0.51 | p = 0.355 |
|  | Lym | (×10^5^/μL) | 10.4 ± 7.28 | 0.42 ± 0.37 | p = 0.001 |
|  | Neu | (×10^5^/μL) | 0.02 ± 0.08 | 0.01 ± 0.10 | p = 0.403 |
