# Supplementary material for: The pronounced lung lesions developing in LATY136F knock-in mice mimic human IgG4-related lung disease
Source: PLoS One. 2021 Mar 4;16(3):e0247173. doi: 10.1371/journal.pone.0247173 (PMC7932159; doi:10.1371/journal.pone.0247173)
Supplement: Attachment — Submitted filename: ResponseToRevewers 20210112.docx [file pone.0247173.s002.docx]

January 12, 2021

Joerg Heber, Editor-in-Chief

*PLOS ONE*

Dear Dr. Heber:

We wish to re-submit our manuscript titled “The pronounced lung lesions developing in *LAT^Y136F^* knock-in mice mimic human IgG4-related lung disease”.

We thank you and the reviewers for your thoughtful suggestions and insights. The manuscript has benefited from these insightful suggestions. I look forward to working with you and the reviewers to move this manuscript closer to publication in *PLOS ONE.*

The manuscript has been rechecked, and the necessary changes have been made in accordance with the reviewers’ suggestions. The responses to all comments are given below.

Thank you for your consideration. I look forward to hearing from you.

Sincerely,

Yuko Waseda

Third Department of Internal Medicine, Faculty of Medical Sciences, University of Fukui, 23-3 Matsuoka Shimoaizuki, Eiheiji, Fukui, Japan, 910-1193

TEL: +81-776-61-8355

FAX: +81-776-61-8111

E-mail: yuwaseda@gmail.com

RESPONSE TO REVIEWER’S COMMENTS

Reviewer #1: The manuscript “The pronounced lung lesions developing in LATY136F knock-in mice mimic human IgG4-related lung disease” by Waseda et al is a concise report that correlated mice that express an alternate form of LAT and the spontaneous lymphoproliferative disorder they develop to human IgG4-related disease. The authors find that these mice exhibit strong Th2 inflammation early in life, followed by fibrosis. This study is quite straight-forward, but would benefit from more detail in many places.
Concerns:
1. This study uses knock-in of LAT in which tyrosine 136 is substituted with a phenylalanine, thereby preventing downstream signaling. This results in drastic lymphoproliferation. Is there any evidence that LAT is involved in human disease? The authors also discuss lung involvement in the introduction and mention clinical characteristics, but these characteristics are not defined. More detail about the clinical observations in this disease and how they correlate to what is seen in the animal model would be helpful.

**COMMENT: 1**

Thank you for your comment. First, whether LAT is related to human disease is unclear. The characteristics of lung lesions in human IgG4-RD were described in the Introduction. The pathological findings include high levels of IgG and IgG4 in serum, infiltration of IgG4-positive plasma cells, obliterative phlebitis or obliterative arteritis and lymphocytes, as well as a form of fibrosis called storiform fibrosis in some organs, such as the pancreas, salivary glands, kidney, retroperitoneum, and periaorta. We found that the lungs of LAT mice also had high serum IgG and IgG1 levels and had histopathology similar to that of humans. Clinical findings include many allergic diseases, such as rhinitis, conjunctivitis, bronchial asthma, and urticaria, but we have not yet investigated whether LAT mice have allergic diseases. Furthermore, in humans, IgG4-RD involves HLA-DRB1 and FCGR2B at the general level (Reference 15), but whether it is related to LAT has not been investigated. We believe that this is also something that needs further consideration in the future, and this manuscript did not consider it to that extent.

2. The authors briefly mention the Th2 nature of their observations and the potential link with allergic disease, but there is no follow-up on this in either the experiments or in the discussion. Are there differences in IgE in this model?

**COMMENT: 2**
Thank you for your advice. As shown in P5L3, one of the characteristics of IgG4-RD is the presence of allergic disease, and IgE often increases. In reference 11, the median IgE of IgG4-related lung disease in 18 patients was 367 (34-2560) U/mL. In addition, IgE is increased in LAT mice (Reference 18). In this study, we revealed that Th2 cytokines are also predominant in the lungs. We believe that this model, including our previous reports, represents the clinical conditions of IgG4-RD. We did not mention it this time, but we will look into presence of allergic disease in LAT mice in future studies.

3. Figure 1 needs more detail in the results and figure legend, especially regarding the 20 weeks sections. It seems that the image on the left is H&E staining and the image on the right is not, but this is unclear and the stains for each section needs to be defined.

**COMMENT: 3**
Thank you for your comment. The image on the right is also H-E staining. The left lung was bleeding, so it looks different from the right lung, but it was similar in terms of bronchodilation etc.

4. The Ashcroft score needs to be defined.

**COMMENT: 4**

We have added the requested information to the text (P10L6).

5. In general, Figures 1-3 would benefit from greater details in the results section.

**COMMENT: 5**

We have added more details to the text describing Figure 1-3.

Reviewer #2: Dr. Wasada et al present a well argued manuscript describing the use of a LAT knock-in mouse as a model of IgG4-RD of the lung. This is a well executed follow-up study of their prior work, published in this journal, establishing this same mouse model in other organs commonly affected by human IgG4 disease. While the authors provide a compelling argument, there are a few minor issues that I suspect, if addressed, would improve the author's argument.
1) Figure 1: you present two LAT 20w x20 figures at the bottom with largely different appearances. Does this speak to the heterogeneity of the results at 20w? If so, would it help to show cumulative data, compiling the fibrosis scores. If this is true heterogeneity, it may be beneficial to add actual datapoints to Figure 3B, 4, and 5 instead of just showing the median.

**COMMENT: 1)**
Thank you for your advice. The image on the left looks different because the lung was bleeding, but it is similar if you disregard the effect of bleeding. Because some mice bleed during fibrosis progression, we showed some lungs that are bleeding and some that are not.

2) Redundancy: the statement, "In addition, in Lat knock in mice, the score significantly increased over time." This is already shown in the sentence above, just with data re-arranged. I would re-word or eliminate this.

**COMMENT: 2)**
Thank you for your advice. The previous sentence shows that the Ashcroft score of LAT mice is significantly higher than that of WT mice after 6 weeks, and the next sentence, "In addition, in Lat knock in mice, the score significantly increased over time.", is a comparison between LAT mice and states that they are different over time.

3) I'm assuming you chose to study BALF at 8,9 weeks due to your prior results suggesting peak inflammation occurred between 6-10 weeks. I wonder if you would have found greater cytokine production at an earlier time-point. It would have been interesting to see the composition of the lymphocytes found in the BALF. This could be done fairly easily using flow cytometry and could provide some insight into the unique cytokine pattern you found with bead array.

**COMMENT: 3)**

Thank you for your fruitful advice. Unfortunately, we have not examined cytokines earlier than 8-9 weeks, but in future experiments, we will use flow cytometry to examine the temporal changes in cytokines.

4) Next steps. Now that you have established this model, how do you intend to study it further?

**COMMENT: 4)**

Thank you for your comment. IgG4-RD is considered to be an allergic disease. Therefore, we would like to examine whether LAT mice have any allergic diseases and evaluate whether inflammation and fibrosis are improved by using antibody preparations such as anti-IL-4, IL-5, and IL-13 antibodies. I think these will be effective for the treatment of IgG4-RD.

5) Wording. There are some phrases throughout the manuscript that are hard to follow, or do not seem to add anything to your argument. For example, on page two, "Regarding the lungs, in addition to comprehensive diagnostic criteria, diagnostic criteria unique to the lungs were established, and diagnosis of lung lesions is performed based on the clinical characteristics." The clarity of your message could be improved greatly by a grammatical review focused on reducing redundancy.

**COMMENT: 5)**

Thank you for your comments. Regarding the lung, IgG4-positive plasma cells may infiltrate the lung even in lungs with collagen disease (we added reference 12), and patients with diseases other than IgG4-related lung disease will be included based on comprehensive diagnostic criteria alone. Therefore, we established diagnostic criteria unique to the lungs. We rewrote the text to suggest that lung lesions can be accurately diagnosed based on clinical findings, pathological findings, and imaging findings. We also had the entire sentence proofread by a native English-speaking scientist.
